# A new role for tamoxifen in oestrogen receptor-negative breast cancer when it is combined with epigallocatechin gallate

**DOI:** 10.1038/sj.bjc.6604634

**Published:** 2008-09-16

**Authors:** M J Scandlyn, E C Stuart, T J Somers-Edgar, A R Menzies, R J Rosengren

**Affiliations:** 1 Department of Pharmacology and Toxicology, University of Otago, Dunedin, New Zealand

**Keywords:** EGCG, tamoxifen, MDA-MB-231, CYP1B1, mTOR, EGFR

## Abstract

We have previously shown that tamoxifen+epigallocatechin gallate (EGCG) is synergistically cytotoxic towards oestrogen receptor (ER)-negative breast cancer cells. To determine if this response would correlate with significant tumour suppression *in vivo*, athymic nude female mice were implanted with MDA-MB-231 cells and treated with tamoxifen, EGCG, EGCG+tamoxifen, or vehicle control for 10 weeks. Tumour volume in EGCG- (25 mg kg^−1^)+tamoxifen (75 *μ*g kg^−1^)-treated mice decreased by 71% as compared with vehicle control (*P*<0.05), whereas tumour weight was decreased by 80% compared with control (*P*<0.01). Epigallocatechin gallate treatment did not alter ER protein expression in MDA-MB-231 cells and thus was not a mechanism for the observed tumour suppression. However, western blotting of tumour extracts demonstrated that epidermal growth factor receptor (EGFR; 85% lower than control), pEGFR (78% lower than control), mammalian target of rapamycin (mTOR; 78% lower than control), and CYP1B1 (75% lower than control) were significantly lower after the combination treatment as compared with all other treatments. Nuclear factor-*κ*B (NF-*κ*B), b-Raf, p-MEK, S6K, 4EBP1, Akt, vascular EGFR-1 (VEGFR-1) and VEGF expressions were decreased in control but not in the individual treatments, whereas MEK, phospholipase D 1/2, TGF*α*, and ERK expressions were not changed after any treatment. The results demonstrate that tamoxifen at realistic doses (75 *μ*g kg^−1^) can suppress the growth of ER-negative breast cancer when combined with EGCG. In addition, the dominant mechanism for tumour suppression is the concomitant decrease in tumour protein expressions of mTOR and the EGFR.

Tamoxifen has been extensively used in the treatment of oestrogen receptor (ER)-positive breast cancer and was recently approved for chemoprevention in high-risk women (Fisher *et al*, 1998, 2005). In ER-positive breast cancer, tamoxifen acts as an SERM, as it antagonises the binding of estradiol to the ER. However, tamoxifen is not a highly selective drug and it elicits pro-apoptotic effects in ER-negative breast cancer cells by the modulation of various cell signalling proteins in an ER-independent manner (Fattman *et al*, 1998; Ferlini *et al*, 1999; Mandlekar *et al*, 2000). However, these effects have generally been reported following relatively high concentrations of tamoxifen. An appealing strategy would be to use tamoxifen in combination with other agents to enhance its action in ER-negative breast cancer. This concept has been shown by combining docetaxel, genistein, black cohosh, and palm oil tocotrienols (Ferlini *et al*, 1997; Guthrie *et al*, 1997; Tanos *et al*, 2002; Bollig *et al*, 2005; Al-Akoum *et al*, 2007) with tamoxifen, which elicited cytotoxicity, apoptosis, and G1 arrest in various ER-negative breast cancer cell lines. Recently, this has been shown in both MDA-MB-231 cells and a xenograft model by combining tamoxifen with OSU-03012, a phosphoinositide-dependent protein kinase-1/Akt inhibitor (Weng *et al*, 2008). Our laboratory has also used ER-negative breast cancer cells to demonstrate that a combination of epigallocatechin gallate (EGCG) and tamoxifen elicits synergistic cytotoxicity (Chisholm *et al*, 2004). We further demonstrated that EGCG+tamoxifen elicits an earlier and enhanced apoptotic response in MDA-MB-231 cells (Stuart *et al*, 2007), and this correlated with a decrease in protein expression of the active form of the epidermal growth factor receptor (EGFR) after combination treatment (Stuart and Rosengren, 2007).

The EGFR is a clinically significant target for ER-negative breast cancer, as it is overexpressed in ER-negative breast cancer cells (Roos *et al*, 1986) as well as in patients with ER-negative breast cancer (Biswas *et al*, 2004). Furthermore, it is involved in cell proliferation, apoptosis, angiogenesis, tumour invasiveness, and distant metastases (Grandis and Sok, 2004). The EGFR exerts its effects on cellular function through the activation of numerous downstream signalling pathways, including the Raf–MEK–ERK mitogen-activated protein kinase pathway and the PI_3_K–Akt pathway (Dutta and Maity, 2007). The stimulation of the Raf–MEK–ERK cascade leads to the activation of numerous transcription factors that are important for cell proliferation and survival (Brott *et al*, 1993). Epidermal growth factor receptor signalling through the PI_3_K–Akt pathway results in the activation of the transcription factor nuclear factor-*κ*B (NF-*κ*B) (constitutively active in ER*α*− breast cancers) and mammalian target of rapamycin (mTOR), an important regulator of protein synthesis (Averous and Proud, 2006). Recently, the importance of the dual suppression of both EGFR and mTOR expressions was shown when the proliferation of breast cancer was synergistically reduced after the inhibition of both EGFR and mTOR expressions (Buck *et al*, 2006).

The aims of this study were to determine whether the tumour suppression potential of the combination of EGCG+tamoxifen in a xenograft model of ER-negative breast cancer. On the basis of our previous *in vitro* studies in MDA-MB-231 cells, we postulated that the inhibition of both EGFR and mTOR expressions would be important mechanisms for tumour suppression after combination treatment. Therefore, we measured EGFR and its major downstream targets in tumours to demonstrate drug-mediated effects on critical cell signalling pathways from treated mice. We also examined tumour protein expression of CYP1B1, because this protein provides a new target for the suppression of tumour growth, as it is overexpressed in many human cancers, including breast cancer (Murray *et al*, 1997).

## Materials and methods

### Chemicals and reagents

Epigallocatechin gallate (99% purity) was purchased from the Cayman Chemical Company (Ann Arbor, MI, USA). MDA-MB-231 human breast cancer cells were purchased from ATCC (Manassas, VA, USA). Matrigel and the primary antibodies Akt, pAkt, EGFR, pEGFR and CYP1B1 were purchased from BD Biosciences (Auckland, New Zealand). Minimum essential medium, potassium chloride, NaHCO_3_, NaCl, trizma hydrochloride, Triton X-100, trypsin, and trypan blue were purchased from Sigma Chemical Co. (St Louis, MO, USA). Acetic acid, disodium hydrogen orthophosphate anhydrous, EDTA, DMSO, potassium dihydrogen orthophosphate, and sodium citrate were purchased from BDH Laboratory supplies (Poole, UK). Primary antibodies NF-*κ*B, bRaf, TGF*α*, 4EBP1, p4EBP1, vascular endothelial growth factor receptor (VEGF), VEGFR-1, mTOR, PLD1, PLD2, S6K, pS6K, MEK, pMEK, ERK, pERK, ER*α*, and ER*β* were purchased from Abcam (Cambridge, MA, USA). Foetal bovine serum was purchased from Life Technologies Ltd. (Auckland, New Zealand). All other chemicals were the highest purity commercially available.

### Cell culture

MDA-MB-231 and MCF-7 cells were maintained in minimum essential medium supplemented with 10% foetal bovine serum, 1% antibiotic/antimitotic solution, and 0.2% NaHCO_3_. Cells were cultured in 75 cm^2^ flasks and incubated in 5% CO_2_/95% humidified air at 37°C.

### Animals and treatment

Female CD1 athymic nude mice (5- to 6-week old) were purchased from Hercus Taieri Resource Unit (Dunedin, New Zealand). They had free access to sterilised standard Reliance Rodent Diet (Dunedin, New Zealand) and water, and were housed four mice per cage and maintained at 21–24°C with a 12-h light/dark cycle in a specifically designed pathogen-free isolation facility and allowed to acclimatise for 1 week before experimentation. The University of Otago Animal Ethics Committee approved all procedures. Mice were inoculated into the right flank with ER-negative cells (MDA-MB-231, 2 × 10^6^/50 *μ*l matrigel), which were left to form palpable tumours. Tumour volume was measured weekly with electronic calipers (L W H). When the tumour volume reached 100 mm^3^, mice (10 per group) were randomly assigned to the various treatment groups. Mice were then treated daily with tamoxifen (50 or 75 *μ*g kg^−1^), EGCG (25 mg kg^−1^), EGCG+tamoxifen, or a vehicle control for each drug (5 ml kg^−1^) for 10 weeks. Dosing solutions were prepared fresh each day. Tamoxifen was given through oral gavage, whereas EGCG was given intraperitoneally. On the account of extensive first-pass metabolism (Li *et al*, 2001), EGCG is often administered parentally (Liao *et al*, 1995; Landis-Piwowar *et al*, 2007; Somers-Edgar *et al*, 2008). In addition, the dose of EGCG (25 mg kg^−1^, i.p.) was based on our previous study, which demonstrated that this dose was non-toxic and elicited a modest decrease in MDA-MB-231 tumour growth (Somers-Edgar *et al*, 2008). This dose is also twofold lower than that recently used in other ER-negative xenograft experiments with EGCG (Landis-Piwowar *et al*, 2007; Thangapazham *et al*, 2007). On the basis of our *in vitro* experiments with EGCG+tamoxifen, we wanted to use a very low dose of tamoxifen. Therefore, we chose a dose of tamoxifen that was at least 10-fold lower than that has typically been used in other xenograft studies.

### Assessment of animal health

Food consumption and body weight were monitored daily throughout the treatment period. Mice were euthanised by CO_2_ inhalation 24 h after the last dose and necropsies were then performed. Blood was collected through the inferior vena cava and placed on ice, whereas major organs, as well as tumours, were excised and weighed. Organ weights are expressed as a percentage of body weight. Plasma was separated and used to determine the presence of hepatotoxicity via the plasma marker alanine aminotransferase activity using a commercially available kit (Medica Pacifica, Auckland, New Zealand). Results are expressed as IU l^−1^.

### Preparation of whole cell lysates

MDA-MB-231 and MCF-7 cells were plated (200 000 cells per well) in six-well plates and treated for 24 h with EGCG (100 *μ*M) or estradiol (100 nM). Cells were harvested using a cell scraper, washed, and incubated in RIPA buffer (50 mM Tris base, 1% NP-40, 0.25% sodium deoxycolate, 100 mM sodium chloride, 1 mM EDTA, and complete protease inhibitor cocktail tablets; Roche, Mannheim, Germany), incubated on ice for 15 min, and centrifuged at 13 000 r.p.m. for 10 min at 4°C. The supernatant was collected and the protein concentration was determined using the BCA assay.

### Western blotting of proteins from cell lysates or tumour extracts

Protein extraction from tumour tissue was performed as described (Somers-Edgar *et al*, 2008). The extracts or cell lysates were fractioned by SDS–PAGE, electrotransferred to nitrocellulose membranes, blotted with each antibody, and detected by amplified colour reagent (Bio-Rad, Auckland, New Zealand). *β*-Actin was used as a loading control, and the density of each band was normalised to the *β*-actin control. For clarity in the figures, densitometry is shown only for proteins that were modulated by drug treatment.

### Immunohistochemistry of tumour sections

Tumours were embedded in OCT compound and then sectioned (5 *μ*m), fixed in acetone, and air-dried overnight. Sections were then washed twice in Tween 20–PBS, incubated with normal serum for 30 min at room temperature, and then incubated overnight with primary CYP1B1 or VEGFR-1 antibody. Slides were then rinsed and peroxidase blocked using hydrogen peroxide (3%) before incubation with the appropriate secondary antibody for 30 min at room temperature in a humidified chamber. The sections were then incubated with ExtrAvidin (Bio-Rad, Auckland, New Zealand) (1 : 20) for 30 min at room temperature in a humidified chamber before development with 3,3′-diaminobenzidine tetrahydrochloride as the chromogen and counterstaining with Mayer's haematoxylin. Once slides were dehydrated, DPX mounting medium and coverslips were applied. The sections were analysed from tumours obtained from each mouse and a representative slide is shown in the results section.

### Statistical analysis

For all parameters where the variances were significantly different between control and treated mice (namely, tumour volume and tumour weight), the data were log-transformed before statistical analysis was performed. Tumour growth experiments were analysed using a repeated measures two-way ANOVA coupled with a Student–Newman–Keuls *post hoc* test, in which *P*<0.05 was the minimum requirement for a statistically significant difference. Analyses that did not involve time (i.e., tumour weight and protein expression) were analysed using an one-way ANOVA coupled with a Student–Newman–Keuls *post hoc* test, in which *P*<0.05 was the minimum requirement for a statistically significant difference.

## Results

### The combination of EGCG and tamoxifen suppresses tumour growth *in vivo*

**Figure 1:**
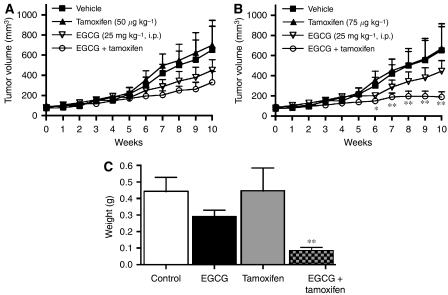
Tumour growth after the treatment with EGCG and tamoxifen. Female athymic nude mice were implanted with MDA-MB-231 cells (2 × 10^6^) and treated for 70 days. (**A**) Tumour volume during the treatment with vehicle (5 ml kg^−1^), EGCG (25 mg kg^−1^, i.p.), tamoxifen (50 *μ*g kg^−1^, p.o.), or EGCG+tamoxifen. (**B**) The tumour volume during treatment with vehicle (5 ml kg^−1^), EGCG (25 mg kg^−1^, i.p.), tamoxifen (75 *μ*g kg^−1^, p.o.), or EGCG+tamoxifen. (**C**) Tumour weight after 70 days of treatment with EGCG (25 mg kg^−1^, i.p.) and tamoxifen (75 *μ*g kg^−1^, p.o.). Symbols and bars represent the mean±s.e.m. from 10 mice per group. ^*^Combination treatment significantly different from vehicle control (*P*<0.05). ^**^Combination treatment significantly different from all other groups (*P*<0.05).

When EGCG (25 mg kg^−1^) was combined with tamoxifen (50 *μ*g kg^−1^), the growth rate of the tumours in the combination treated mice lagged behind the other treatments, but an overall significant decrease in tumour growth was not observed (Figure 1A). A significant decrease in tumour growth was observed when the dose of tamoxifen in combination with EGCG was increased to 75 *μ*g kg^−1^. Specifically, after 6 weeks of treatment, the growth curve from the combination treated mice diverged from other treatments (Figure 1B). After 10 weeks of treatment, the combination treated mice had a tumour volume that was 71.3% smaller than both the vehicle- and tamoxifen-treated mice (*P*<0.05). Tumour weight was also decreased significantly after combination treatment (tumour weights of 0.45±0.08, 0.45±0.14, 0.29±0.4 and 0.09±0.2 g for vehicle, tamoxifen, EGCG and EGCG+tamoxifen, respectively) (Figure 1C). Importantly, the drug treatments were well tolerated and non-toxic to the mice, as weight gain and all other physiological parameters (i.e., liver, kidney, spleen and uterine weight) were in the normal range (data not shown) and no hepatotoxicity was observed as alanine aminotransferase activity was not elevated following any of the drug treatments (values ranged between 23 and 27 IU l^−1^).

### EGCG does not reverse the ER status of MDA-MB-231 breast cancer cells

**Figure 2:**
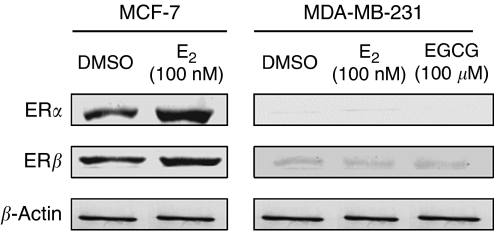
Protein expressions of ER*α* and ER*β* in MCF-7 and MDA-MB-231 cells. Cells were treated with 100 nM of estradiol (E_2_) or with 100 *μ*M of EGCG for 24 h. The blots shown are representative of three independent experiments performed in duplicate.

As EGCG has upregulated ER*α* expression in cell lines with a low ER*α* expression (Belguise *et al*, 2007), it was necessary to determine whether the tumour suppression could be due to an EGCG-mediated change in the protein expression of the ER in MDA-MB-231 cells. Therefore, MDA-MB-231 cells were treated with EGCG (100 *μ*M) or estradiol (100 nM) for 24 h, and MCF-7 cells were treated with estradiol (100 nM) as a control. The results demonstrated that estradiol increased the protein expression of both ER*α* and ER*β* in MCF-7 cells, whereas neither EGCG nor estradiol induced the protein expression of the ER in MDA-MB-231 cells (Figure 2).

### The combination of EGCG and tamoxifen regulates EGFR and mTOR *in vivo*

**Figure 3:**
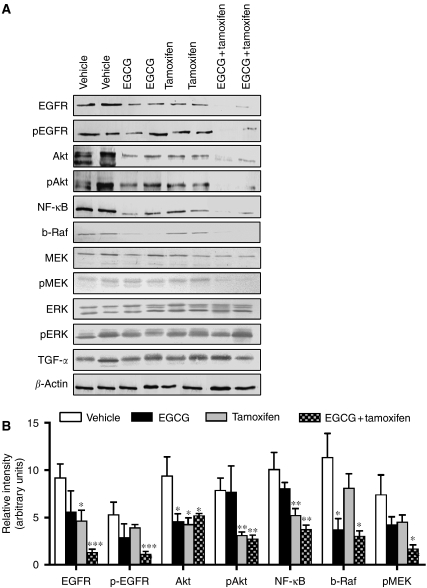
Tumour protein expression levels of EGFR, Akt, NF-*κ*B, b-Raf, ERK and MEK after treatment with EGCG and tamoxifen. Mice were treated with vehicle (5 ml kg^−1^), EGCG (25 mg kg^−1^, i.p.), tamoxifen (75 *μ*g kg^−1^, p.o.), or EGCG+tamoxifen for 70 days. (**A**) Representative western blots of the various proteins from each individual mouse. (**B**) The results of scanning densitometry of western blots. For clarity, densitometry is shown only for proteins that were modulated by drug treatment. Bars represent the mean±s.e.m. from 10 mice per group. ^*^Significantly different from vehicle control (*P*<0.05). ^**^Significantly different from vehicle and EGCG (*P*<0.01). ^***^Significantly different from all other treatments (*P*<0001).

As we have previously shown that EGCG+4-OHT produces an earlier and greater apoptotic response (Stuart *et al*, 2007) and elicits a greater inhibition of pEGFR in MDA-MB-231 cells (Stuart and Rosengren, 2007), the role of the EGFR and other downstream cell signalling proteins were determined in tumour extracts. The results showed that the protein expression of EGFR was significantly decreased in tumours from combination-treated mice as compared with all other treatments (relative density of EGFR in the EGCG+tamoxifen group was decreased by 85.3±3.33, 76.0±5.5 and 71.2±6.6% as compared with control, EGCG, and tamoxifen, respectively, *P*<0.001) (Figure 3A and B). A similar trend was also observed for the active form of EGFR pEGFR (relative density of pEGFR in the EGCG+tamoxifen group was decreased by 78.4±5.3, 71.2±7.0 and 61.0±9.6% as compared with control, tamoxifen, and EGCG, respectively, *P*<0.001) (Figure 3A and B). However, TGF*α*, a regulator of EGFR signalling, was not changed following any of the treatments (Figure 3A). Given that the combination of EGCG+tamoxifen significantly reduced levels of both EGFR and pEGFR, downstream targets of this receptor were examined. As the EGFR signals predominantly through the mitogen-activated protein kinase cascade and the PI_3_K–Akt pathways, components of these pathways were investigated. Protein expression of the mitogen-activated protein kinase cascade members MEK, ERK, and pERK were unchanged following any of the treatments (Figure 3A). However, combination treatment caused tumour protein levels of the ERK regulator pMEK to decrease by 76.6±5.3% as compared with vehicle control (*P*<0.05) (Figure 3A and B). Other downstream targets measured (Akt, NF-*κ*B, and b-Raf) in tumours from combination-treated mice were all significantly decreased in control, whereas p-Akt and NF-*κ*B were also decreased in control and EGCG (Figure 3A and B).

**Figure 4:**
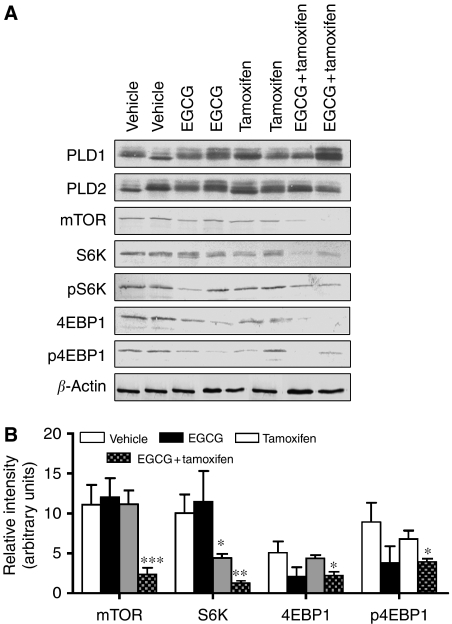
Tumour protein expression levels of mTOR, S6K and 4EBP1 after treatment with EGCG and tamoxifen. Mice were treated with vehicle (5 ml kg^−1^), EGCG (25 mg kg^−1^, i.p.), tamoxifen (75 *μ*g kg^−1^, p.o.), or EGCG+tamoxifen for 70 days. (**A**) Representative western blots of the various proteins from each individual mouse. (**B**) The results of scanning densitometry of western blots. For clarity, densitometry is shown only for proteins that were modulated by drug treatment. Bars represent the mean±s.e.m. from 10 mice per group. ^*^Significantly different from vehicle control (*P*<0.05). ^**^Significantly different from vehicle and EGCG (*P*<0.01). ^***^Significantly different to all other treatments (*P*<0001).

As mTOR is a major regulator of protein translation and other processes important in cell proliferation, this protein as well as its targets, S6K and 4EBP1 were also examined in tumour extracts. The results demonstrated that protein expression of mTOR and S6K were significantly decreased in tumours from combination treatment mice as compared with all other treatment groups (relative density of mTOR was decreased by 78.0±6.9, 78.4±6.8 and 80.0±6.3% as compared with control, tamoxifen, and EGCG, respectively, *P*<0.001) (Figure 4A and B). However, pS6K was not changed following any of the treatments, whereas both 4EBP1 and p4EBP1 were decreased in control but not in the individual treatments (Figure 4A and B). Phospholipase D (PLD) 1 and 2 represent an alternate upstream regulator of mTOR. However, the protein expression of these isoforms was not altered by any of the treatments (Figure 4A).

### The combination of EGCG and tamoxifen regulates VEGF, VEGFR-1, and CYP1B1 *in vivo*

**Figure 5:**
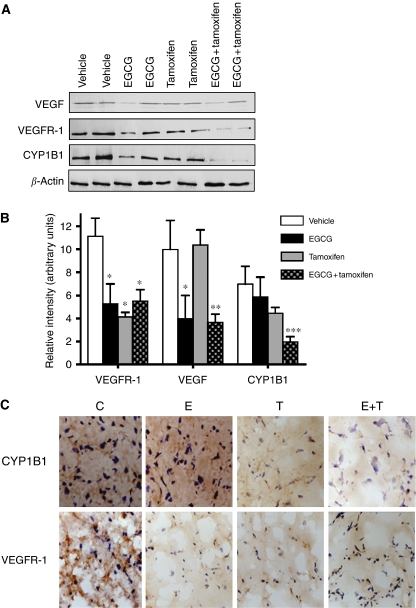
Tumour protein expression levels of VEGF, VEGFR-1, and CYP1B1 after treatment with EGCG and tamoxifen. Mice were treated with vehicle (5 ml kg^−1^), EGCG (25 mg kg^−1^, i.p.), tamoxifen (75 *μ*g kg^−1^, p.o.), or EGCG+tamoxifen for 70 days. (**A**) Representative western blots of the various proteins from each individual mouse. (**B**) The results of scanning densitometry of western blots. For clarity, densitometry is shown only for proteins that were modulated by drug treatment. Bars represent the mean±s.e.m. from 10 mice per group. (**C**) Representative immunohistochemistry of CYP1B1 and VEGFR-1 from tumour sections of treated mice. ^*^Significantly different from vehicle control (*P*<0.05). ^**^Significantly different from vehicle and tamoxifen (*P*<0.01). ^***^Significantly different from all other treatments (*P*<0001).

The tumour marker CYP1B1 was also decreased by 71.5% in tumour tissue from combination-treated mice as compared with vehicle control as shown by western blotting and immunohistochemistry (Figure 5A–C). This protein was also decreased as compared with each single drug treatment (relative intensities of 1.98±0.42, 4.47±0.48, and 5.88±1.69 for EGCG+tamoxifen, tamoxifen, and EGCG, respectively, *P*<0.05). To gain insight into the role of angiogenesis in the growing tumour, VEGF, an important regulator of angiogenesis (Sledge, 2002), which also aids in breast cancer cell survival (Wu *et al*, 2006), was examined in tumour extracts. All treatments decreased both VEGF and its receptor VEGFR-1 approximately 50% from control as shown by both western blotting and immunohistochemistry (Figure 5A–C), but this effect was not enhanced after combination treatment.

## Discussion

We have shown that the combination of tamoxifen (75 *μ*g kg^−1^, p.o.) and EGCG (25 mg kg, i.p.) inhibits the growth of MDA-MB-231 xenografts and that this effect is superior to that elicited by either individual treatment. Another study has shown that the combination of green tea extract (2.5 g/l) and tamoxifen (20 mg pellet) is better than either drug alone at suppressing the growth of MCF-7 xenografts, and this correlated with increased levels of apoptosis and a suppression of angiogenesis in tumour tissue (Sartippour *et al*, 2006). However, this drug combination has not previously been examined in an *in vivo* model of ER-negative breast cancer.

Our results demonstrated that tamoxifen on its own was not effective at suppressing ER-negative tumour growth, whereas EGCG had a modest effect on tumour growth, but the combination of the two drugs showed marked tumour suppression. Other studies have also shown that EGCG or green tea extract has a modest inhibitory effect on ER-negative tumour growth (Landis-Piwowar *et al*, 2007; Thangapazham *et al*, 2007; Somers-Edgar *et al*, 2008). For example, EGCG (50 mg kg^−1^, s.c.) was minimally effective at reducing tumour growth in a MDA-MB-231 xenograft model, but acetylating EGCG and thus turning it into a pro-drug enhanced its efficacy (Landis-Piwowar *et al*, 2007). In a similar xenograft model, the efficacy of EGCG was increased by the addition of curcumin (Somers-Edgar *et al*, 2008). However, the overall tumour volume was only decreased by 49% as compared with control after EGCG+curcumin treatment, indicating that EGCG+tamoxifen is a more effective combination therapy as tumour volume was decreased by 71% as compared with control. Another recent study has shown that ER-negative breast cancer cells can be sensitised to the effects of tamoxifen by using a PDK-1/Akt inhibitor (Weng *et al*, 2008). In this study, tamoxifen (60 mg kg^−1^) was unable to suppress the growth of MDA-MB-231 xenografts, but the addition of OUS-03012 (100 mg kg^−1^) showed significant tumour suppression (decreased 50% in control). This result supports the use of tamoxifen in combination therapies for ER-negative breast cancer. However, our combination with EGCG produced a greater level of tumour suppression at a much lower dose of tamoxifen (75 *μ*g kg^−1^).

The mechanism responsible for the enhanced tumour suppression and apoptosis (Stuart *et al*, 2007) elicited by EGCG+tamoxifen does not involve EGCG-mediated changes in the ER status of MDA-MB-231 cells. Furthermore, when the ER is present, tamoxifen acts to antagonise the effects of estradiol, and as our model does not involve the use of estradiol pellets, there is no ER-mediated response to antagonise. Instead, the mechanism is most likely to be initiated by the modulation of EGFR activity. Our results demonstrate that both protein levels of EGFR and its active phosphorylated form are significantly reduced by EGCG+tamoxifen treatment. Although there was no further reduction in the active form of EGFR after combination treatment, the similar decrease in both the unphosphorylated and phosphorylated forms indicates that the signalling capacity of the pathway as a whole was reduced by approximately 78% by the combination treatment. Combination treatment also caused a similar reduction in the protein expression of mTOR. The inhibition of mTOR is of particular significance, as this is a key regulator of protein synthesis within the cell. Furthermore, the growth of renal cell carcinoma (Gemmill *et al*, 2005), glioblastoma multiforme (Goudar *et al*, 2005), as well as non-small-cell lung cancer, pancreatic, colon, and breast (Buck *et al*, 2006) cancers were synergistically reduced when EGFR inhibitors were combined with the mTOR inhibitor rapamycin. The *in vitro* results correlated with synergistic tumour suppression in a xenograft model of non-small-cell lung cancer. Interestingly, the results showed neither of the individual drugs significantly suppressed tumour growth, but showed pronounced reduction after combination treatment. Our results are very similar, in that pronounced reduction in tumour growth only occurred after EGCG+tamoxifen. As a similar (∼80%) and significant reduction of EGFR and mTOR occurred after combination therapy, it is most likely that this dual suppression is a key mechanistic component of the observed tumour suppression. Furthermore, the decreased expression of mTOR is not likely to be regulated further upstream through PLD1/2, as levels of PLD1 and PLD2 were not altered by any drug treatments. However, the combined inhibition of EGFR, Akt, and mTOR after EGCG+tamoxifen treatment provides a powerful suppression of tumour growth.

Studies with EGCG in a variety of models have demonstrated that it decreases angiogenesis (Cao and Cao, 1999; Sartippour *et al*, 2001, 2002). Our results support this, as tumour-derived VEGF was decreased after EGCG treatment. Therefore, the inhibition of VEGF after combination treatment appears to be mediated by EGCG, and thus the inhibition of tumour-derived VEGF is not a mechanism for the combination treatment. However, VEGF and VEGFR-1 levels in the surrounding mouse stromal tissue would provide additional information regarding the overall effect of the drug treatment on angiogenesis.

Another important effect elicited by the drug combination is the 78% decrease in the protein expression of CYP1B1. CYP1B1 has not been examined previously following the *in vivo* administration of EGCG. However, this protein could be important in ER-negative breast cancer, as the *CYP1B1*_1358_A>G polymorphism has been significantly associated with ER-negatvie tumour status (Justenhoven *et al*, 2008). This genotype is known to encode higher CYP1B1 activity and it is believed to contribute to low levels of the ER in these tumours by a reduction in estradiol. As EGCG has been shown to modulate CYP1B1 in MCF-7 cells (Guengerich *et al*, 2003) and antagonise AhR-mediated responses (Palermo *et al*, 2005), our results of decreased CYP1B1 protein expression after combination treatment is not surprising. The role of CYP1B1 in tumour suppression was also shown in a study where CYP1B1 null mice were protected against DMBA-induced tumours as compared with their wild-type counterparts (Buters *et al*, 1999). As CYP1B1 can inactivate the prostate cancer dug flutamide (Rochat *et al*, 2001) and is also induced in breast cancer cells after treatment with docetaxel (Martinez *et al*, 2008), it may have an important function in the development of resistance to chemotherapy. Overall, CYP1B1 has a variety of functions in both the treatment and development of breast cancer. Therefore, the 78% inhibition of CYP1B1 elicited by EGCG+tamoxifen treatment is a critical component of its mechanism of action.

In summary, we have demonstrated that the combination of EGCG+tamoxifen significantly reduced ER-negative tumour growth and that this was driven primarily by an enhanced effect by the decreased protein expression of the EGFR, mTOR, and CYP1B1. Overall, our findings suggest that EGCG+tamoxifen as an antitumorigenic therapy in ER-negative breast cancer is promising and thus this combination warrants further investigation.
